# Comparison of reducing effect on lung injury of dexamethasone and bosentan in acute lung injury: an experimental study

**DOI:** 10.1186/2049-6958-8-74

**Published:** 2013-12-17

**Authors:** Omer Araz, Elif Demirci, Elif Yilmazel Ucar, Muhammet Calik, Didem Pulur, Adem Karaman, Muhammed Yayla, Eren Altun, Zekai Halici, Metin Akgun

**Affiliations:** 1 Department of Pulmonary Diseases, Ataturk University School of Medicine, Erzurum, Turkey; 2 Department of Pathology, Ataturk University School of Medicine, Erzurum, Turkey; 3 Department of Pulmonary Diseases, Zonguldak Government Hospital, Zonguldak, Turkey; 4 Department of Radiology, Ataturk University School of Medicine, Erzurum, Turkey; 5 Department of Pharmacology, Ataturk University School of Medicine, Erzurum, Turkey; 6 Chest Disease Department, Yakutiye Medical Research Center, 25240 Erzurum, Yakutiye, Turkey

**Keywords:** Acute lung injury, Bosentan, Dexamethasone

## Abstract

**Background:**

Different medical therapies are employed in acute lung injury (ALI) but there is still a debate about the efficacy of these drugs. Among these therapies steroids are clinically applied and bosentan is experimentally studied. The aim of this study was to evaluate the efficacy of these two drugs to treat inflammation in ALI by histopathological comparison.

**Methods:**

The five experimental groups (n = 5 per group) were: saline control (Group I); lipopolysaccharide (LPS) + saline (Group II); LPS + dexamethasone (Group III); LPS + 50 mg/kg bosentan (Group IV); and LPS + 100 mg/kg bosentan (Group V). Bosentan was administered orally one hour before and 12 hours after LPS treatment. Dexamethasone was administered intraperitoneally in three doses of 1 mg/kg; one dose was co-administered with LPS and the other two doses were given respectively 30 minutes before and after LPS treatment. Vasodilation-congestion, hemorrhage, polymorphonuclear leukocyte (PMN) infiltration, mononuclear leukocyte (MNL) infiltration, alveolar wall thickening, alveolar destruction/emphysematous appearance, and focal organization were the parameters used as criteria for evaluating inflammation and efficacy of treatment.

**Results:**

Compared to the LPS-only group (Group II), dexamethasone treatment (Group III) resulted in significant improvements in vasodilation-congestion, hemorrhage, PMN and MNL infiltration, alveolar wall thickening and emphysematous areas. Treatment with 50 mg/kg dose of bosentan (Group IV) also resulted in significant improvements in hemorrhage, PMN and MNL infiltration, alveolar wall thickening and alveolar destruction. Reducing lung injury and reparative effects of 100 mg/kg bosentan were significant in all parameters.

**Conclusions:**

Bosentan is as effective as dexamethasone for treating lung injury in ALI. Bosentan at 100 mg/kg can be recommended as a first treatment choice based on its significant reducing lung injury and reparative effects.

## Background

Sepsis is a systemic inflammatory response to infection and a major cause of morbidity and mortality worldwide [1]. Sepsis is characterized by progressive development of conditions including systemic inflammatory response syndrome (SIRS), tissue damage and multiple organ dysfunction syndrome (MODS), and acute respiratory distress syndrome or acute lung injury (ARDS/ALI). Although the pathophysiology of sepsis is not well defined, monocytes orchestrate the innate immune response to Gram-positive and Gram-negative bacteria by expressing a variety of inflammatory cytokines, including tumor necrosis factor (TNF)-a and interleukin (IL)-6, which are believed to play an essential role in the pathogenesis of sepsis [2-7].

ARDS/ALI, one of the potential complications of sepsis, is a serious condition with a high mortality rate (30-50%). Acute pulmonary inflammation is mediated by a number of cytokines and reactive oxygen species (ROS) produced by a variety of inflammatory cells. Direct cell damage by ROS is caused by the oxidation of cell membrane lipid moieties. ROS can be produced by activated macrophages, endothelial cells, or polymorphonuclear neutrophils (PMN). The infiltration of PMN is mediated by a chemokine gradient and may be the key event that drives a pulmonary oxidant damage [8].

To date, treatment strategies for ARDS/ALI have shown limited success in improving clinical outcomes, with the exception of low tidal volume mechanical ventilation [9]. Considering the evidence that inflammation contributes to the pathogenesis of ARDS/ALI [10], therapies that attenuate this inflammation – such as corticosteroids, potent anti-inflammatory agents and immunomodulators, which act in multiple stages of the inflammatory cascade [11] – should be investigated. There are two opposing views concerning the use of steroids in treating ARDS/ALI. One criticism is that steroids are ineffective in the early or late management of ARDS/ALI [12]; however, there have been positive reports that the use of steroids significantly increased survival [13]. Thus the role of steroids in the treatment of ARDS/ALI is debatable.

Treatment options of ARDS/ALI also include experimental drugs reducing lung injury similar to corticosteroids. Endothelin-1 (ET-1) receptor antagonist bosentan is one of such drugs. Endothelin has four receptors (ET-A, ET-B1, ET-B2 and ET-C) and bosentan exerts its effect through ET-A and -B receptors [14,15]. ET-1 is a peptide produced by endothelial cells and several studies have demonstrated its important role in lung inflammation. ET-1 has significant pro-inflammatory effects in airways [16,17], whereas endothelin receptor antagonists mitigate its pro-inflammatory effects in animal models of airway inflammation [15,18]. Research into the reducing lung injury of endothelin-1 receptor antagonists is ongoing.

In the current study, histopathologic techniques were used to compare the efficacy of dexamethasone and two doses of bosentan in treating LPS-induced pulmonary inflammation.

## Methods

### Animals

A total of 25 male Wistar rats weighing 220–250 g were used. All rats were obtained from Ataturk University’s Experimental Animal Laboratory of the Medicinal and Experimental Application and Research Center (ATADEM). Animal experiments and procedures were performed in accordance with national guidelines for the use and care of laboratory animals and were approved by Ataturk University’s local animal care committee (442190979-01-02/2831). The rats were housed in standard plastic cages on sawdust bedding in a climate-controlled room (22 ± 1°C). Standard rat food and tap water were provided *ad libitum*.

### Drugs and endotoxin

LPS from *Escherichia coli* serotype 0111:B4 (Sigma-Aldrich Srl, Milan, Italy) was prepared in sterile saline, aliquoted, and stored at -80°C for short periods. All chemicals were purchased from Sigma Chemical Co. (Munich, Germany). Bosentan was obtained from Actelion Pharmaceuticals Ltd (Allschwil, Switzerland).

### Experimental design

#### Experimental groups

Five experimental groups (5 rats per group) were used: vehicle (saline) control (Group I); lipopolysaccharide (LPS) + saline (Group II); LPS + dexamethasone (Group III); LPS + 50 mg/kg bosentan (Group IV); and LPS + 100 mg/kg bosentan (Group V).

#### Drug administration

Bosentan was administered orally one hour before and 12 hours after LPS treatment. Dexamethasone was administered intraperitoneally in three doses of 1 mg/kg; one dose was co-administered with LPS and the other two doses were given 30 minutes before and after LPS treatment. The groups were housed in separate cages.

### Sepsis model

The animals in the vehicle control group (Group I) received 1 ml salineintraperitoneally twice at an interval of 30 minutes. One dose of 1 mg/kg LPS was administeredintraperitoneally to the animals in Groups II, III, IV and V. All animals were monitored for 24 hours; none of them perished during the observation period. All animals were sacrificed after 24 hours by 50 mg/kg thiopental sodium injection. The lungs were removed immediately and washed with ice-cold saline. Tissues were fixed in 10% formalin for histopathological analysis.

### Histological procedure

#### Histochemical methods

Rat pulmonary tissue samples were fixed in 10% formalin for 2–4 days and embedded in paraffin. Sections of 4 micron thickness were taken and stained with hematoxylin-eosin. Sections were evaluated by two independent pathologists with an Olympus BX51 microscope according to the following parameters: vasodilation-congestion, hemorrhage, polymorphonuclear leukocyte (PMN) infiltration, mononuclear leukocyte (MNL) infiltration, alveolar wall thickening, alveolar destruction/emphysematous appearance and areas of focal organization. Parameters were assessed using a 4-point grading system: Grade 0 = none; Grade 1 (+) = mild; Grade 2 (++) = moderate; Grade 3 (+++) = severe.

#### Immunohistochemical methods

Immunohistochemistry was performed using a Leica Bond-max automated immunostainer (Leica Microsystems, Newcastle, UK), as described by the manufacturer’s protocol. The prepared 4-μm tissue sections were deparaffinized in a dry oven, dewaxed in xylene and rehydrated through graded alcohol. Heat pretreatment was performed in citrate buffer (pH 6.0) at 100°C for 20 minutes. Sections were treated with peroxide for 5 minutes, then anti-Endothelin 1 antibody (ab49591, rabbit monoclonal, 1:50; Abcam, Cambridge, UK) was applied for 30 minutes. Antibody binding was detected using a bond polymer refine kit (Leica Microsystems) and diaminobenzidinetetrahydrochloride solution (Kit HK153-5 K; Biogenex, San Ramon, CA, USA) was used as a chromogen.

The analysis was done using an Olympus BX51 microscope and the two analyzing pathologists were blind regarding the group from which the sections belonged. The staining proportion score was determined by percent of cells stained: <10% stained = 1 (low); 10-50% stained = 2 (medium); ≥50% = 3 (high). The numerical value for staining intensity was determined on a 3-point scale: 1, 2, 3 (for light, medium and dark staining).

### Statistical analyses

The data were analyzed using SPSS version 17 statistical software (SPSS Inc., Chicago, IL). One-way ANOVA followed by Tukey’s test was used to analyze the inflammation within groups. P < 0.05 was considered statistically significant.

## Results

### Histopathological results

**Figure 1:**
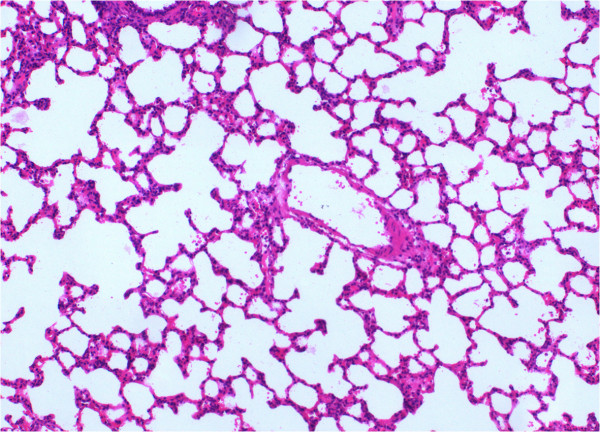
H&E stained sections from saline control (Group I) (×100).

**Figure 2:**
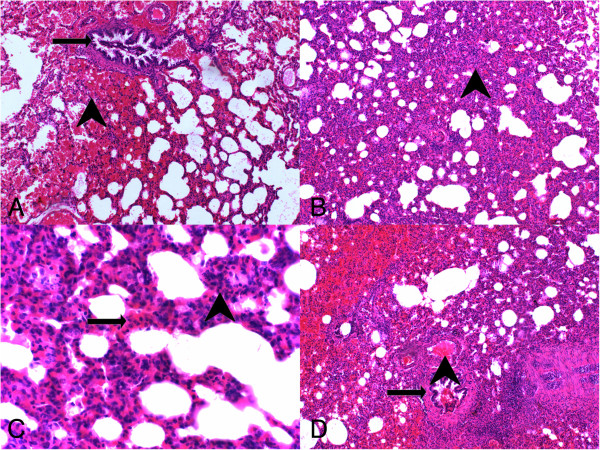
**H&E stained sections from LPS-only group. (A)** arrowhead, hemorrhage, arrow, terminal bronchiole (H&E, ×100); **(B)** arrowhead, thickening of the interalveolar septum, alveolar filling defects (H&E, ×100), **(C)** arrowhead, PNL infiltration, arrow, MNL infiltration (H&E, ×200), **(D)** arrowhead, vasodilation-congestion, arrow, terminal bronchiole (H&E, ×100).

**Figure 3:**
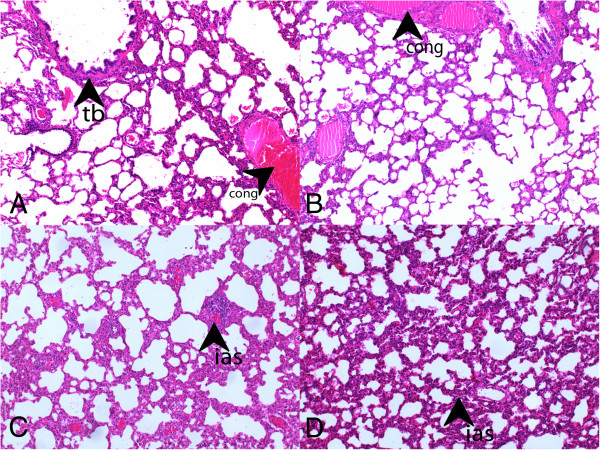
**H&E stained sections from Group III (dexamethasone) and IV (50 mg/kg bosentan).** (**A**, **B**: Group III) tb, terminal bronchiole, cong, congestion (H&E, ×100), (**C**, **D**: Group IV) ias,interalveolar septum (H&E, ×100).

**Figure 4:**
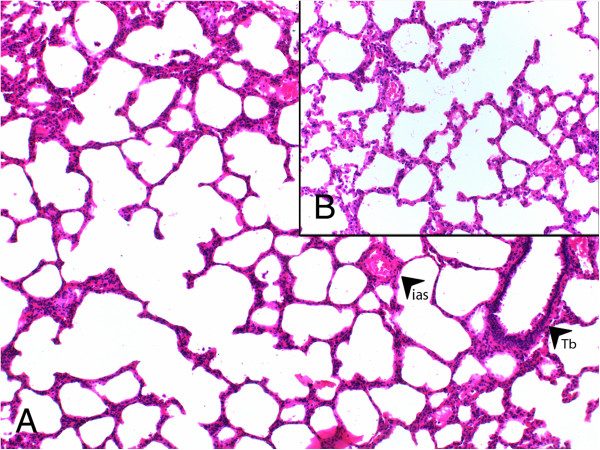
**H&E stained sections from Group V (100 mg/kg bosentan). (A)** ias, interalveolar septum, Tb, terminal bronchiole (×100), **(B)** (×200).

Group I had the lowest scores in all parameters evaluated in histochemical analyses (Figure 1). From all experimental groups, Group II (LPS-only) had the highest scores and most severe presentation in the parameters of vasodilation-congestion, hemorrhage, PMN and MNL infiltration, alveolar wall thickening and emphysematous areas (Figure 2). When compared to Group II, treatment with dexamethasone (Group III), 50 mg/kg bosentan (Group IV) (Figure 3) and 100 mg/kg bosentan (Group V) each produced significant improvements in most parameters. The amelioration of vasodilation-congestion was most significant with 100 mg/kg bosentan (Group V). The attenuation of inflammatory reaction (PMN and MNL infiltration) and resolution of hemorrhage were similar between 100 mg/kg bosentan and dexamethasone. The reversal of focal organization was most marked with 100 mg/kg bosentan (Figure 4). In general, 50 mg/kg bosentan effected improvement compared to LPS-only treatment, but this improvement was not as pronounced as with dexamethasone or 100 mg/kg bosentan.

### Immunohistochemical results

**Figure 5:**
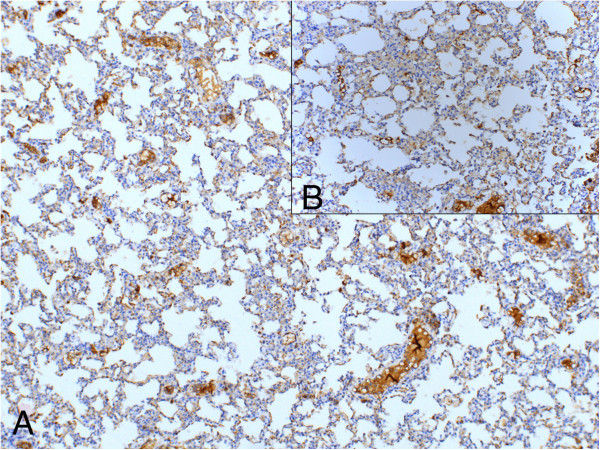
**Proportion and intensity of anti-endothelin staining in sections from Group V (100 mg/kg bosentan). (A)** anti-endothelin (LM, ×100); **(B)** anti-endothelin (LM, ×200).

**Figure 6:**
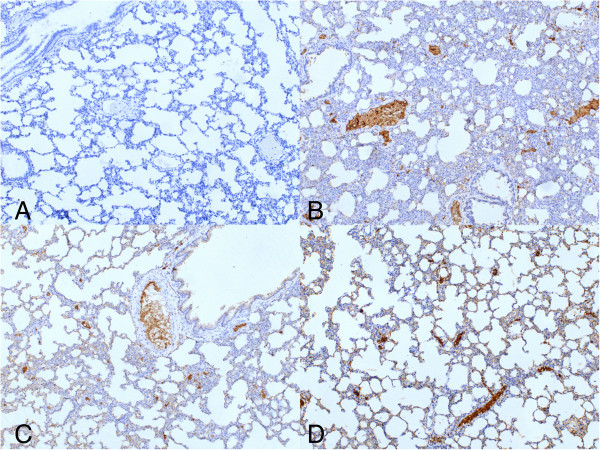
**Proportion and intensity of anti-endothelin staining in sections from Groups I-IV (LM, ×100). (A)** Group I (saline control); **(B)** Group II (LPS-only); **(C)** Group III (dexamethasone); **(D)** Group IV (50 mg/kg bosentan).

In immunohistochemical analyses, 100 mg/kg bosentan showed the highest staining proportion and intensity scores, followed by 50 mg/kg bosentan and dexamethasone (Figure 5). Anti-endothelin-1 reactivity in the LPS-only group (Group II) was higher than in controls (Group I), but it was markedly lower compared to the treatment groups (Groups III-V) (Figure 6).

### Clinical results

**Figure 7:**
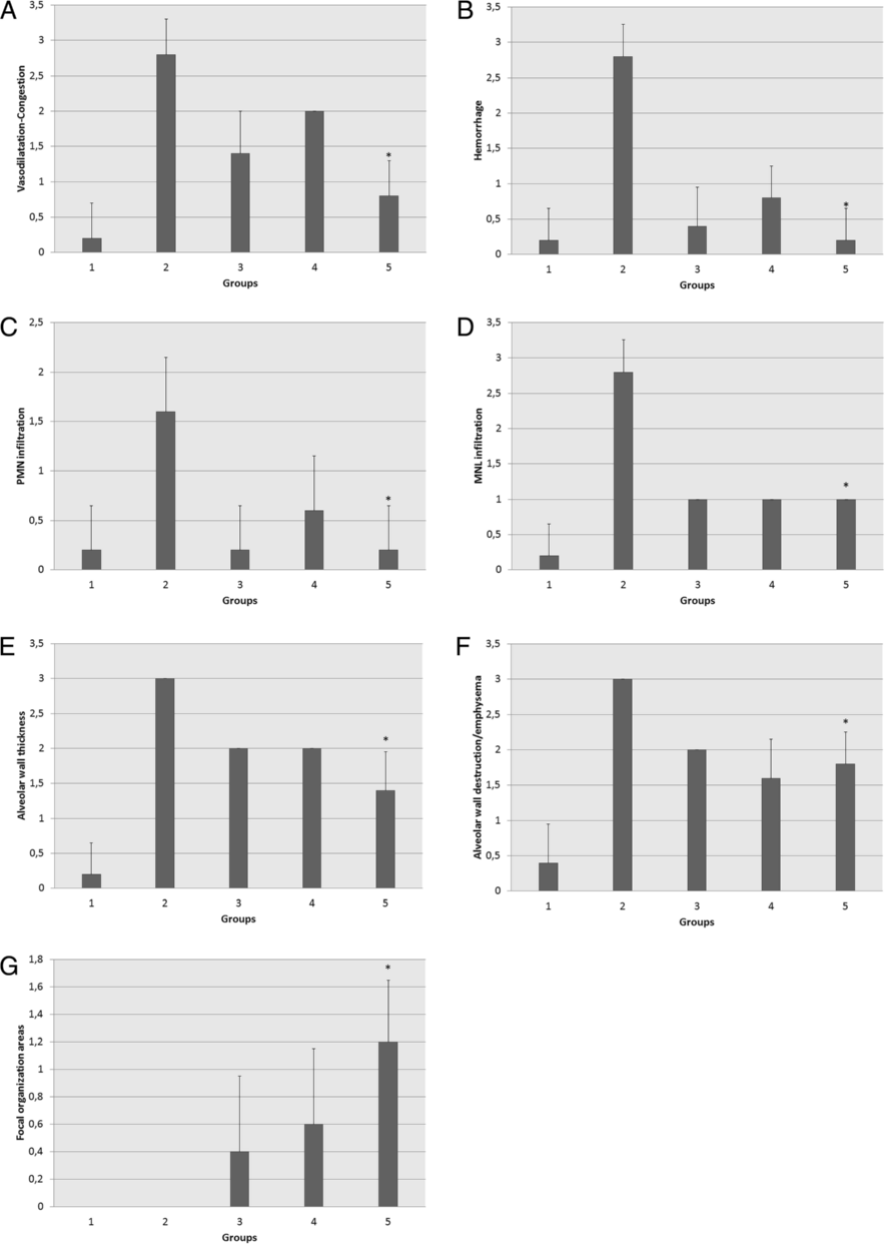
**Comparison of vasodilation-congestion (A) hemorrhage (B) polymorphonuclear (PMN) leukocyte infiltration (C) mononuclear leukocyte (MNL) infiltration (D) alveolar wall thickening (E) alveolar destruction-emphysematous appearance (F) and focal organization between Group II (LPS-only) and Group V (100 mg/kg bosentan).** *p = 0.0001 for **A**, **B**, **D**, **E** and **F**, p = 0.002 for **C**, and p = 0.001 for **G**.

Treatment with dexamethasone (Group III) resulted in significant improvements in vasodilation-congestion (p = 0.0001), hemorrhage (p = 0.0001), PMN (p = 0.002) and MNL (p = 0.0001) infiltration, alveolar wall thickening (p = 0.001) and emphysematous areas (p = 0.0001) compared to the LPS-only group. Treatment with 50 mg/kg bosentan (Group IV) also significantly mitigated hemorrhage (p = 0.0001), PMN (p = 0.031) and MNL (p = 0.0001) infiltration, alveolar wall thickening (p = 0.001) and alveolar destruction (p = 0.01). The reducing lung injury and ameliorative effects of 100 mg/kg bosentan were significant in all parameters (p = 0.0001) (Figure 7A-G).

When comparing parameters of reducing lung injury between treatment groups (Groups III-V), focal organization was the only statistically significant difference between dexamethasone and 100 mg/kg bosentan treatments (p = 0.035). Vasodilation-congestion was the only parameter significantly different between 50 and 100 mg/kg bosentan treatment (p = 0.002).

**Figure 8:**
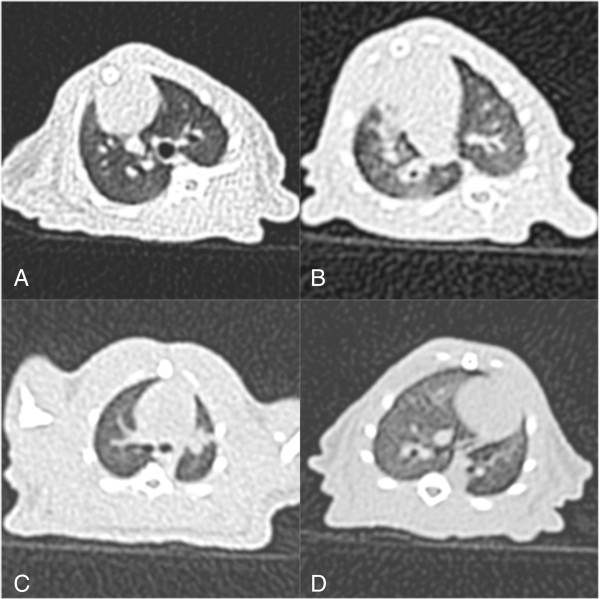
(A) Unenhanced Thoracic CT scan shows lung parenchyma (A) LPS-induced ARDS model showing diffuse alveolar opacities (B) characteristic bilateral diffuse airspace consolidations with a marked anteroposterior gradient; bilateral peripheral areas of hyperlucency representing trapped air are also apparent (C) bilateral reduction in alveolar opacities compared to the LPS-only group (D).

A computed tomography (CT) scan was performed on a representative sample of one animal from each of the following groups: saline control (Figure 8A), LPS-only (Figure 8B), dexamethasone (Figure 8C) and 100 mg/kg bosentan (Figure 8D).

## Discussion

In this study, dexamethasone was shown to reduce lung injury in an animal model of ALI; compared to dexamethasone, whereas bosentan demonstrated equal or greater efficacy. Although the reducing lung injury of bosentan at 50 mg/kg and 100 mg/kg was similar, the latter dose caused greater improvement in focal organization, which indicates overall repair. Furthermore, compared to the other treatment groups, 100 mg/kg bosentan generally effected the most significant improvement in all parameters and resulted in the highest staining proportion and intensity scores of all experimental groups.

The array of pathophysiological changes induced by LPS challenge resemble those often accompanying Gram-negative bacteria sepsis [19-22]. A common and frequently lethal complication of sepsis is ARDS/ALI, which is associated with pulmonary microvascular injury and is characterized by severe hypoxemia, diffuse lung infiltration, reduction in compliance, and increased pulmonary resistance [19,22,23].

It has been recognized that infiltration and activation of phagocytes are mediating factors in LPS-induced ARDS/ALI. Phagocytes are known to play a key role in lung injury by a cascade effect: phagocytes release oxidants and proteases that damage the pulmonary endothelial and epithelial cells, thus disrupting the alveolar-capillary unit [24,25]. Glucocorticoids such as dexamethasone are used in the treatment of inflammatory lung injuries due to their ability to inhibit phagocyte migration to the site of injury and to partially suppress phagocyte reactivity [26]. Dexamethasone pretreatment has also reduced pulmonary elastase activity and chloramine levels, further supporting the anti-inflammatory effectiveness of dexamethasone. [27] In this study, dexamethasone treatment produced marked reducing lung injury in all observed parameters (vasodilation-congestion, hemorrhage, PMN and MNL infiltration, alveolar wall thickening, alveolar destruction-emphysematous appearance). These findings support the use of dexamethasone as a treatment for ARDS/ALI.

Though not applied clinically, the endothelin receptor antagonist bosentan is used to treat ARDS/ALI experimentally. Although endothelin is commonly associated with pulmonary hypertension due to its vasoconstrictive effect, it is also a vasoactive peptide that acts as pro-inflammatory agent, stimulating the release of cytokines and production of reactive oxygen species by PMN [28]. A previous study demonstrated that bosentan treatment significantly reduced the production of reactive oxygen species by PMN during acute lung inflammation [29]. Another study demonstrated diminished levels of the pro-inflammatory cytokines TNFα, interleukin-1, interleukin-6 and interleukin-8 in rat pulmonary tissue following bosentan treatment of induced emphysema [30]. Both doses of bosentan used in the current study demonstrated pronounced reduction of lung injury. Amelioration of vasodilation-congestion was more significant with 100 mg/kg bosentan rather than with 50 mg/kg. The 100 mg/kg dose of bosentan showed the strongest overall reduction of lung injury among all the treatment groups; this may be attributable to the strong attenuation of vasodilation, which facilitates cell migration. Furthermore, 100 mg/kg bosentan caused significant reduction of focal organization, which indicates amelioration of inflammation. These findings support the efficacy of 100 mg/kg bosentan as reducing lung injury agent.

Despite the known effects of endothelin receptor antagonists on inflammation in lungs, their impact on the production of reactive oxygen species by PMN following induction of acute lung inflammation is not known [31]. In the current study, endothelin receptor staining using anti-endothelin-1 antibody was performed; staining proportion and intensity were measured to represent prevalence and quantity of endothelin receptor. These values and PMN and MNL infiltration data were analyzed to assess the effect of endothelin receptor on PMN-MNL migration. Treatment with 100 mg/kg bosentan resulted in higher anti-endothelin-1 staining proportion and intensity compared to dexamethasone, but its effect on PMN-MNL migration was similar, suggesting that prevalence and quantity of the endothelin receptor antigen does not affect PMN-MNL migration. However, 100 mg/kg bosentan had the most significant effect on both focal organization and receptor prevalence and quantity, which suggests that an elevated level of endothelin receptor contributes to ameliorating inflammation.

## Conclusions

In conclusion, reducing lung injury of bosentan is comparable to that of dexamethasone in the treatment of ALI. Bosentan at 100 mg/kg may be preferable as a first treatment option due to its significant reducing lung injury and ameliorating effects. However, larger clinical trials of bosentan are needed.

## Competing interests

The authors received no financial support for the research and/nor authorship of this article. The authors declare that they have no competing interest to the publication of this article.

## Authors’ contributions

OA, Assistant Professor Doctor, design of the study. ED, Assistant Professor Doctor, data analysis. EYU, Assistant Professor Doctor, acquisition of data. MC, Assistant Professor Doctor, data analysis. DP, Specialist Doctor, statistical analysis. AK, Assistant Professor Doctor, data analysis. MY, Assistant Doctor, acquisition of data. EA, Assistant Doctor, acquisition of data. ZH, Associate Professor Doctor, design of the study. MA, Professor Doctor, FCCP, editing of the manuscript. All authors read and approved the final manuscript.
